# A Dictionary of Terms Used in Medicine and the Collateral Sciences

**Published:** 1845-11

**Authors:** 


ART. XII.—*A Dictionary of Terms used in Medicine and the Collateral Sciences.* By RICHARD D. HOBLYN, A.M., Oxon. First American from the second London edition. *Revised, with numerous additions*, by ISAAC HAYS, M.D., Editor of the American Journal of the Medical Sciences. Philadelphia: Lea & Blanchard. 1845. 12mo. pp. 402.

We have given the dimensions of this Dictionary as constituting one of its principal claims to public favor. It is concise, and so may be made the constant companion of the student, and being small is also cheap. The fact that it has passed under the revision of Dr. Hays, is a guaranty that it contains everything that might be looked for in such a work. We take it to be specially adapted to the wants of medical students during their attendance upon lectures, to whom its portable form and small cost will be strong recommendations. Dr. Hays, by various additions and alterations, has adapted it to the American reader.

